# The effect of ellagic acid on the repair process of periodontal defects related to experimental periodontitis in rats

**DOI:** 10.1590/1678-7757-2021-0160

**Published:** 2021-09-27

**Authors:** Figen ÖNGÖZ DEDE, Şeyma BOZKURT DOĞAN, Umut BALLI, Mustafa Cenk DURMUŞLAR, Bahattin AVCI, Kanat GÜLLE, Meryem AKPOLAT FERAH

**Affiliations:** 1 Ordu University Faculty of Dentistry Department of Periodontology Ordu Turkey Ordu University, Faculty of Dentistry, Department of Periodontology, Ordu, Turkey.; 2 Yıldırım Beyazıt University Faculty of Dentistry Department of Periodontology Ankara Turkey Yıldırım Beyazıt University, Faculty of Dentistry, Department of Periodontology, Ankara, Turkey.; 3 Bezmialem Vakif University Faculty of Dentistry Department of Periodontology İstanbul Turkey Bezmialem Vakif University, Faculty of Dentistry, Department of Periodontology, İstanbul, Turkey.; 4 Kent University Faculty of Dentistry Department of Oral and Maxillofacial Surgery İstanbul Turkey Kent University, Faculty of Dentistry, Department of Oral and Maxillofacial Surgery, İstanbul, Turkey.; 5 Ondokuzmayis University Faculty of Medicine Department of Biochemistry Samsun Turkey Ondokuzmayis University, Faculty of Medicine, Department of Biochemistry, Samsun, Turkey.; 6 Süleyman Demirel University Faculty of Medicin Department of Histology and Embryology Isparta Turkey Süleyman Demirel University, Faculty of Medicine, Department of Histology and Embryology, Isparta, Turkey.; 7 Bülent Ecevit University Faculty of Medicine Department of Histology and Embryology Zonguldak Turkey Bülent Ecevit University, Faculty of Medicine, Department of Histology and Embryology, Zonguldak, Turkey.

**Keywords:** Ellagic acid, Periodontitis, Periodontal repair, Cytokines, Antioxidant, Alveolar bone defect

## Abstract

**Objective:**

This study aims to evaluate the effect of ellagic acid (EA) by measuring the levels of alveolar bone resorption and inflammatory and oxidative stress markers in the periodontal tissues and serum on the periodontal repair process related to experimental periodontitis in rats.

**Methodology:**

Forty Wistar rats were divided into four study groups as follows: Group 1=healthy control (n=10); Group 2=EA control (15 mg/kg)(n=10); Group 3=periodontitis (n=10); Group 4=periodontitis+EA (15 mg/kg) (n=10). The periodontitis model was established by ligating bilateral mandibular first molars for 14 days. Then, rats were given normal saline or EA for another 14 days by gavage administration. Serum and gingiva myeloperoxidase (MPO) activity, 8-hydroxydeoxyguanosine(8-OHdG), and glutathione (GSH) levels were analyzed by ELISA. İmmunohistochemical analysis was used to detect Interleukin (IL)-6, IL-10, and tumor necrosis factor-alpha (TNF-α) immunoreactivities in the periodontal tissues. Alveolar bone loss (ABL) and attachment loss (AL) was evaluated by histomorphometry analysis.

**Results:**

ABL and AL were statistically higher in group 3 than in groups 1, 2 and 4 and in group 4 than in groups 1 and 2 (p<0.05). MPO activities in gingival tissue and serum were significantly increased in group 3 compared to groups 1 and 2 (p<0.05). Significantly higher serum GSH levels, lower gingiva, and serum 8-OHdG levels, and MPO activity were observed in group 4 compared to group 3 (p<0.05). Rats with periodontitis (group 3) expressed significantly higher immunoreactivities of IL-6 and TNF-α and lower IL-10 immunoreactivity compared to those other groups (p<0.05). IL-6 and TNF-α immunoreactivities significantly decreased and IL-10 immunoreactivity increased in group 4 after the use of EA compared to group 3 (p<0.001).

**Conclusions:**

Our findings showed that EA provides significant improvements on gingival oxidative stress and inflammatory markers and alveolar bone resorption in the repair process associated with experimental periodontitis. Therefore, EA may have a therapeutic potential on periodontitis.

## Introduction

Periodontitis is one of the most widespread infectious inflammatory diseases. It occurs due to an imbalance between dental plaque bacteria and the host’s inflammatory and immune responses.^1^ During periodontitis, host cells produce excessive amounts of reactive oxygen species (ROS), such as those produced by myeloperoxidase (MPO), 8-hydroxydeoxyguanosine (8-OHdG) and inflammatory cytokines, such as tumor necrosis factor-alpha (TNF-α) and interleukin (IL)-6, in response to bacterial infiltration. The release of these markers leads to the destruction of periodontal tissues.^2–4^

The elimination of microbial dental biofilms is essential to the treatment of periodontal diseases.^1^^,^^5^ Mechanical removal (scaling and root planning) is not sufficient for the complete elimination of periodontal microflora.^1^^,^^5^ Therefore, pharmacologic agents (antiseptics, nonsteroidal anti-inflammatory drugs, and antibiotics) are used as adjunctive therapy to ensure the destruction of microorganisms.^6^ When these drugs are used systemically with high doses, they can lead to complications such as antibiotic resistance and other side effects.^1^ This is the reason why alternative natural products are needed for therapeutic use.^1^^,^^6^^,^^7^ For such purpose, polyphenolic compounds, which have antioxidant, anti-cancer, and anti-inflammatory effects have been suggested as potential candidates.^8^^,^^9^ Polyphenols have been reported to have several biological activities; they prevent oral disease, inactivate bacterial toxins, promote the antioxidant activity of oral fluids, exhibit antibacterial activity against periodontal pathogens, and can inhibit the proteolytic activity of *Porphyromonas gingivalis.*^3^^,^^9^^,^^10^

Ellagic acid (EA), a polyphenol, is found in many fruits, including strawberries, walnuts, pomegranates, and grapes, and many medicinal plants.^11^^,^^12^ EA has been reported to exhibit antioxidant, anti-cancer, anti-allergic, antiproliferative, and anti-inflammatory activities, as well as radical scavenging activity and inhibition of lipid peroxidation.^11–14^ Ogawa, et al.^15^ (2002) reported that EA eliminated superoxide and hydroxy anions. They stated that EA’s effect was stronger than that of α-tocopherol and it was as potent as superoxide dismutase (SOD). EA has been suggested to suppress the production of various cytokines, such as IL-1β, IL-8, and TNF-α.^15^ A recent review concluded that EA could be a promising agent for the treatment of various chronic diseases, especially Alzheimer’s disease, ulcerative colitis, Crohn’s disease, and diabetes.^16^ Moreover, Promsong, et al.^17^ (2015) showed that EA protects human gingival epithelial cells by reducing IL-2 and IL-8 levels and suppressing defense factors, such as human beta-defensin 2 (hBD2) and secretory leukocyte protease inhibitor (SLPI). Bakkiyaraj, et al.^7^ (2013) showed that the anti-biofilm activity of EA was greater in various bacterial pathogens (Staphylococcus aureus, methicillin-resistant S. aureus [MRSA] compared to *Candida albicans.*

Considering these observations, since ROS and inflammatory cytokines are involved in the pathogenesis of periodontitis, we theorize that EA exhibits its anti-inflammatory and antioxidant effects in periodontal lesions by scavenging radicals and suppressing cytokine production. However, no studies have investigated the effect of EA in periodontitis. Therefore, our study aimed to evaluate the therapeutic effects of EA administered orally, measuring gingiva and serum MPO, 8-OHdG, and glutathione (GSH) levels, expression of IL-6, IL-10, and TNF-α in gingiva and alveolar bone loss on repair process associated with experimental periodontitis (EP) in rats.

## Methodology

### Animals

Forty systemically and periodontally healthy adult (8 weeks old) male Wistar albino rats weighing an average of 220 to 250 kg were used in our study. Rats were placed in separate plastic cages, provided food and water being *ad libitum*, housed at a room temperature of 22±1°C within 50% humidity conditions in a 12-hour light/dark cycle. All animal care and experimental protocols were approved by the Ethical Committee of Animal Research of Bulent Ecevit University in Zonguldak, Turkey (Protocol number 2013-29-02/10) in accordance with both the Guide for Care and Use of Laboratory Animals (National Institute of Health, Bethesda, MD, USA) and the ARRIVE guidelines.^18^

Sample size could be estimated before the study due to the lack of precise information available regarding ellagic acid effects in experimental periodontitis. We, therefore, based our estimates on the pilot study, which suggested 6 rats in each group. The sample size was estimated based on the results of biochemical biomarkers levels in gingival tissue between ellagic acid application groups and their control groups. A sample size of 10 per group was required for detection of a significant difference (80% power, two-sided 5% significant level).

The animals were randomly divided into four groups of ten rats each. 1) Group 1=periodontally healthy control (n=10), in which each rat was gavaged daily with 2 mL of saline by gastric intubation for 14 days; 2) Group 2=periodontally healthy rats+ EA control (15 mg/kg)(n=10), in which rat was gavaged daily with 2 mL of saline containing EA by gastric intubation for 14 days; 3) Group 3=experimental periodontitis group (n=10), in which each rat was gavaged daily with 2 mL of saline by gastric intubation for 14 days; 4) Group 4=experimental periodontitis group + EA (15 mg/kg)(n=10), in which rat was gavaged daily with 2 mL of saline containing EA by gastric intubation for 14 days.^19^ The dosage and administration form of drugs were determined based on the literature.^19^ Drug treatment began after periodontitis was induced.

### Experimental periodontitis and repair protocol

A ligature-induced periodontitis model was created due to the induction of EP.^20^ With the rats under general anesthesia through intraperitoneal injection of ketamine (100 mg/kg of body weight) and xylazine1 (10 mg/kg of body weight), EP was induced by the placement of sterile 3–0 silk ligatures in a subgingival position around the mandibular first molars (left and right) in all groups for 14 days, except groups 1 and 2. The ligatures contributed to periodontal diseases, facilitating the movement and passage of bacteria within the gingival cavities.^21^ All ligatures in groups 3 and 4 were removed to allow periodontal repair after 14 days of periodontitis induction^22^. Then, rats started receiving once-daily normal saline or EA for 14 days by gavage administration. The rats were euthanized on the 28^th^ day.

### Treatment with ellagic acid

The EA (Sigma Chemical Company, St Louis, MO, USA) treatment in rats was conducted as described by Kannan, Quine, and Sangeetha^19^ (2012). As soon as EA display poor solubility in water, the treatment in each animal was carried out with a suspension of ellagic in water at a dose of 15mg/kg. The suspension was homogenized with the syringe used in the oral administration before each animal treatment. EA (15 mg\kg) was once-daily administered by gavage for 14 days, starting after the ligature removal in group 4.

### Sample collection

All rats were anesthetized and 5mL of venous blood was drained out through cardiac punctures for serum analyses. Blood samples were centrifuged (Shimadzu UV160A, SNo:28006648, Kyoto, Japan) at 3000 g and room temperature for 10 minutes, enabling the collection of serum, which were then placed at −70°C before biochemical analysis. Block biopsy samples, including the gingiva and alveolar bone tissue, were removed from the molar regions of the mandibles. For histological analysis, the left mandibular molar regions were resected en bloc from each rat and were fixed in 4% paraformaldehyde in 0.1 mol/L phosphate buffer (pH 7.4) for 1 day. Gingival biopsy samples of the right mandibular molar regions were collected and immediately frozen and kept at −70°C until biochemical analysis.

### Biochemical analysis

The gingival tissue was blotted before being weighed upon a microbalance. The tissues were cryogenically frozen using liquid nitrogen. Subsequently, the tissues were manually grounded, by placing them within Eppendorf tubes containing a required volume of PBS (pH 7.4, 10mM), diluted to 10 mg. tissue/mL PBS. This was sonicated (METU Electromechanical, Serial No.30607, Berlin, Germany) for 10 minutes at 4°C with 220V. On the day of evaluation, homogenates defrosted within the room from the samples were centrifuged (SIGMA 3K30, Serial No.76262, Osterode am Harz, Germany) at 4°C and 15000g for 5 minutes and the supernatants were arranged for subsequent GSH, MPO, and 8-OHdG analysis. Gingival tissue and serum MPO activity, GSH, and 8-OHdG levels were evaluated using commercially marketed enzyme-linked immunosorbent assay (ELISA) kits (Cayman Chemical Company, Item No. 589320 Ann Arbor, MI, USA). The quantum of protein present within the tissues was observed by the Lowry method,^23^ with the results expressed as mg per protein. The conclusions were expressed in microgram (µg) of cytokine\mg of protein (µg\mg.protein) within the gingival tissues, and in ng\mL within the serum, barring GSH concentrations. The GSH concentration was expressed as mg\ mg prot within the gingival tissues, and as mg\L within the serum.

### Immunohistochemical analyses

Avidin-biotin peroxidase method was used for the immunohistochemical studies to investigate the anti-IL 10, IL-6, and TNF-α activities. Cross-sections prepared from the mandible tissue blocks in 4 μm thickness were incubated at 60°C. Tissues were rinsed with sequential xylol and alcohol solutions for deparaffinization and distilled water was used to remove the alcohol from dehydrated tissues. To expose the receptor areas within the tissue blocked by formaldehyde, citrate buffer (pH 6.0) (Lab Vision, Fremont, USA) was applied to the tissues under high temperatures. Following the antigen retrieval procedure, the tissues were left in the room to cool down for 20 minutes and then rinsed with distilled water to remove the citrate. Each Tissue was rinsed with phosphate buffer saline (PBS, Ph: 7.4) three times for 3 minutes, exposed to 3% hydrogen peroxide (Lab Vision, Fremont, USA) for 15 minutes, and the endogenous peroxidase activity was blocked and rinsed with PBS. Ultra V block (Lab Vision, Thermo Scientific) was applied for 5 minutes to prevent nonspecific binding. Following the blocking stage, the sections were left at room temperature for 45 minutes without being washed and were exposed to anti-IL-10 antibody (ab9722, Abcam, UK), anti-IL-6 antibody (ab6672, Abcam, UK), anti-TNF-α antibody (NB600-587, Novus biological, USA) and primer antibodies that were prepared 1/100 proportion. All primer antibodies were incubated for an hour at 40ºC. Then they were rinsed with PBS following the primer antibody. A secondary antibody (Lab Vision, Thermo Scientific) was applied for 10 minutes. They were rinsed with PBS and were exposed to streptavidin peroxidase enzyme (Lab Vision, Thermo Scientific) complex for 10 minutes. Each tissue was rinsed once more with PBS. Finally, chromagen DAB (Spring Bioscience) containing the diaminobenzidine substrate (Spring Bioscience) was added to the medium and was put aside for about 5-10 minutes to ensure the immune reaction. Mayer’s hematoxylin was used as the background strain. The slides were rinsed with serial alcohol solutions with diminishing concentrations. They were kept in xylol for 20 minutes and were coated with entallan. All sections were evaluated in the light microscope Axio Scope A1 Imager Microscope (Carl Zeiss, Oberkochen, Germany).

The immunoreactivities of IL-6, IL-10, and TNF-α proteins in the periodontal ligament were scored by one researcher (KG), using a histologic scoring method (HScore).^24^ Staining intensity was semiquantitatively scored according to the following categories: 0, absent; 1, weak; 2, moderate; and 3, intense. The formula was HScore = S Pi (i + 1), in which i represents the intensity scores: Pi, the percentage of stained cells; and 1,the correction factor. In total, 100 positive stained cells in the field were evaluated according to the staining intensity and formulated as follows [1×(%cells1+)+2×(%cells2+)+3×(%cells 3+)].

### Histomorphometric analysis

The left side of the mandible detached from within the gingiva was fixated with 10% neutral buffered formalin. The samples collected were decalcified in 8% formic acid (14 days) and subsequently embedded within paraffin. Serialized paraffin sections (5 µm) were concluded from within the mesiodistal aspects within the mandibular first molars. Three of the sections, reflective of the central parameters of the individual tooth, were observed and thereafter stained with hematoxylin and eosin (H&E).

Individual sections were stained with H&E and the parameters assessed included: 1) the percentage of alveolar bone in the furcation area, 2) alveolar bone loss (ABL), and 3) attachment loss (AL). The percentage ratios of alveolar bone area upon individual specimens were concluded as a ratio of the alveolar bone area versus the furcation area. The alveolar bone area was concluded as a mix of the trabecular bone area and the bone marrow area in furcation. The levels of the alveolar bone were concluded through a measure of the distances within the cementoenamel junction (CEJ) and the alveolar bone crest. AL was concluded to be the distance within the CEJ versus the coronal extent of the connective tissue attached to the cementum. ABL and AL values were obtained from within mesial and distal regions of the mandibular first molars. All averages of the measurements performed were used to analyzing the data.

### Intra-examiner reproducibility

Before histomorphometric and immunohistochemical analysis, the examiner (K.G.), who was blinded to the groups and treatments, evaluated 20 specimens twice, with one-week interval between the measures. Bland-Altman plots along with intraclass correlation coefficients were used to perform the Intra examiner agreement and reliability measures.^25^ Bland–Altman plots reflected the agreements within the two values obtained within one-week interval in the histomorphometric and immunohistochemical parameters.

### Statistical analysis

The Shapiro–Wilk test was used to determine if the data were normally distributed. Comparisons of the biochemical parameters, histomorphometric data, and immunohistochemical scores were analyzed using the Kruskal–Wallis nonparametric test, followed by post-hoc group comparisons with the Bonferroni-adjusted Mann–Whitney U test. The Spearman’s rank correlation test was used to detect the relationships among biochemical parameters, histomorphometric data, and immunohistochemical scores. All tests were performed using statistical software (SPSS Inc., version 19.0, Chicago, IL, USA). p<0.05 was considered statistically significant.

## Results

### Histomorphometric findings

**Figure 1:**
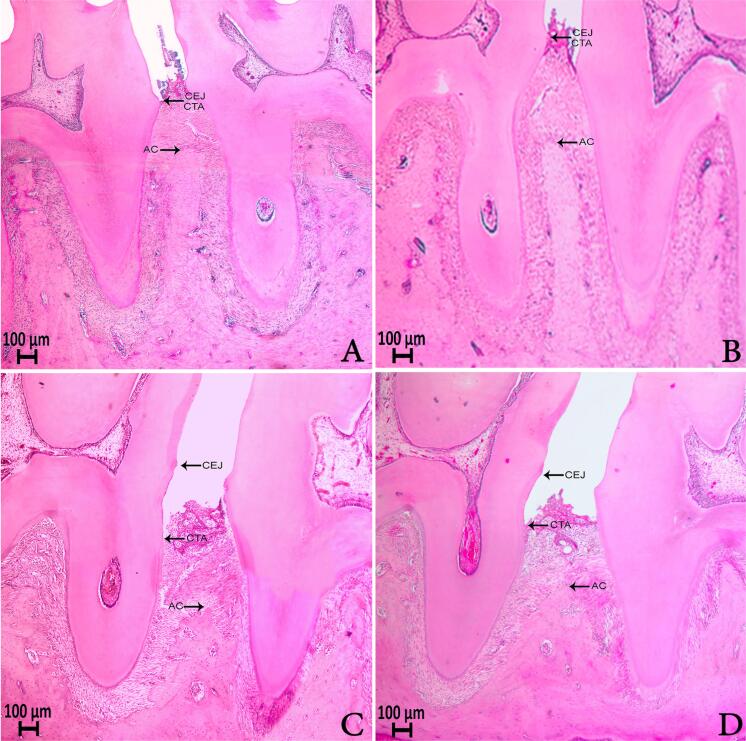
(A) Sections from the mesiodistal aspects throughout the mandibular first molars in the control group (Group 1) (H&E, 4×), (B) Sections from the mesiodistal aspects throughout the mandibular first molars in Group 2 (H&E, 4×), (C) Sections from the mesiodistal aspects throughout the mandibular first molars in Group 3 (H&E, 4×), (D) Sections from the mesiodistal aspects throughout the mandibular first molars in Group 4 (H&E, 4×) CEJ: Cemento-enamel junction, CTA: Connective tissue attachment, AC: Alveolar crest

Table 1 shows the alveolar bone area in the furcation region, alveolar bone level, and attachment loss values. The alveolar bone area of furcation was greater in control groups (p<0.001). However, no significant differences in the amount of alveolar bone in the furcation area in experimental groups were observed (p>0.05). ABL and AL were higher in groups 3 and 4 when compared with groups 1 and 2 (P<0.001). On the other hand, ABL and AL values decreased in group 4 after EA administration than in group 3 (p<0.01). No significant differences in the alveolar bone area of furcation, ABL, and AL values between groups 1 and 2 were observed (p>0.05). Figure 1 shows histologic images.

**Table 1:** Percentage of Alveolar Bone in Furcation Area, Alveolar Bone Level, and Attachment Level

	Groups			
	Group 1 (n=10)	Group 2 (n=10)	Group 3 (n=10)	Group 4 (n=10)
Alveolar Bone Area (%)	64.08±5.57	64.26±4.55	46.70±4.37*^†^	51.47±5.71*^†^
Attachment level (μm)	127.79±18.64	128.64±13.52	525.57±23.64*^†^	407.25±64.30*^†‡^
Alveolar Bone Level (μm)	436.26±21.45	479.19±35.60	1205.47±71.40*^†^	1085.63±90.47*^†‡^

Data are expressed as the mean ± standard deviation.

p<0.05 was considered statistically significant.

Group 1: control-systemic saline, Group 2: control-systemic ellagic acid, Group 3: experimental periodontitis-systemic saline, Group 4: experimental periodontitis-systemic ellagic acid.

* Statistically significant difference from Group 1 (p<0.01).

† Statistically significant difference from Group 2 (p<0.001)

‡ Statistically significant difference from Group 3 (p<0.001)

### Immunohistochemical findings

**Figure 2:**
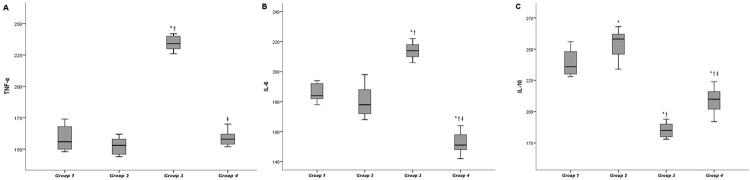
HSCORE Values of IL-10, IL-6 and TNF-α Immunoreactivity in the periodontal ligament for all groups (A) TNF-α immunoreactivity; (B) IL-6 immunoreactivity; (C) IL-10 immunoreactivity Data are expressed as the mean ± standard deviation. p<0.05 was considered statistically significant. Group 1: control-systemic saline, Group 2: control-systemic ellagic acid, Group 3: experimental periodontitis-systemic saline, Group 4: experimental periodontitis-systemic ellagic acid. * Statistically significant difference from Group 1 (p<0.001). † Statistically significant difference from Group 2 (p<0.001). ‡ Statistically significant difference from Group 3 (p<0.01).

Figure 2 shows Immunoreactivity findings of IL-10, IL-6, and TNF-α in gingival tissues. IL-6 and TNF-α immunoreactivities were greater in group 3 among the groups (p<0.001). TNF-α immunoreactivity was statistically decreased after EA administration in group 4 when compared with group 3 (P<0.001). IL-6 immunoreactivity was statistically lower in group 4 than those in other groups (P<0.001).

**Figure 3:**
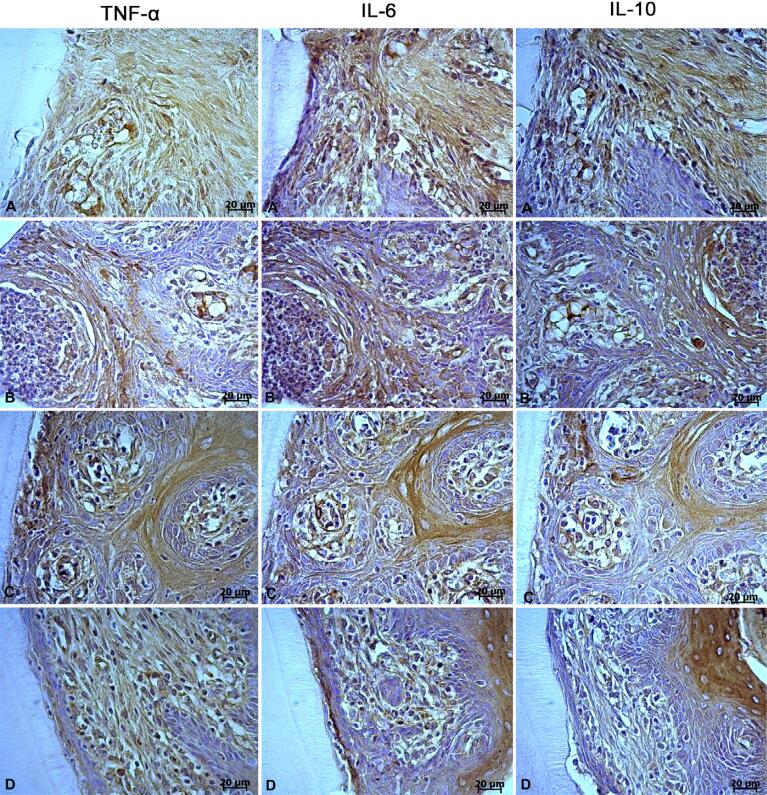
TNF-α, IL-6, and IL-10 immunoreactivity in the periodontal tissues of the rats for all groups. A) Group 1; B) Group 2; C) Group 3; D) Group 4 Group 1: control-systemic saline, Group 2: control-systemic ellagic acid, Group 3: experimental periodontitis-systemic saline, Group 4: experimental periodontitis-systemic ellagic acid.

IL-10 immunoreactivity was significantly greater in group 2 than group 1 (p<0.01), and its level was lower in group 3 than in groups 1, 2, and 4 (p<0.001). Figure 3 shows immunohistochemical images.

### Biochemical findings

**Figure 4:**
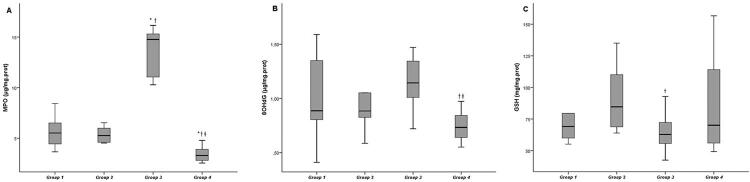
Levels of GSH, MPO, and 8-OHdG in rat gingival tissue Data are expressed as the mean ± standard deviation. p<0.05 was considered statistically significant. Group 1: control-systemic saline, Group 2: control-systemic ellagic acid, Group 3: experimental periodontitis-systemic saline, Group 4: experimental periodontitis-systemic ellagic acid. * Statistically significant difference from Group 1 (p<0.05). † Statistically significant difference from Group 2 (p<0.05). ‡ Statistically significant difference from Group 3 (p<0.05).

**Figure 5:**
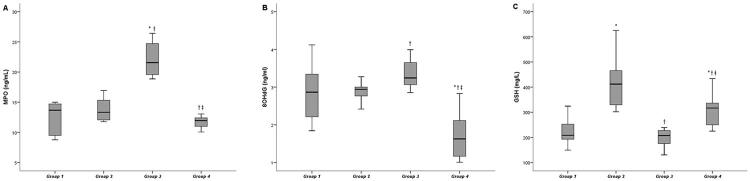
Levels of GSH, MPO, and 8-OHdG in rat serum Data are expressed as the mean ± standard deviation. p<0.05 was considered statistically significant. Group 1: control-systemic saline, Group 2: control-systemic ellagic acid, Group 3: experimental periodontitis-systemic saline, Group 4: experimental periodontitis-systemic ellagic acid. * Statistically significant difference from Group 1 (p<0.05). † Statistically significant difference from Group 2 (p<0.05). ‡ Statistically significant difference from Group 3 (p<0.05).

Serum and gingival tissue MPO activities were higher in group 3 than in groups 1 and 2 (p<0.05). After EA administration in group 4, MPO activities in the gingival tissue and serum significantly decreased when compared with groups 1, 2, and 3 (p<0.05). On the other hand, no significant difference was found in gingival 8-OHdG levels between group 3 and the control groups (p>0.05). However, after EA administration in group 4, 8-OHdG levels in the gingival tissue significantly decreased when compared with groups 2 and 3, and its serum level in group 4 presented the lowest value when compared with those of other groups (p<0.05). EA administration in groups 2 and 4 significantly increased in serum GSH levels compared to groups 1 and 3 (p<0.05), but not in gingival tissue (p>0.05). Figures 4 and 5 show the biochemical findings for gingival tissue and serum values, respectively.

### Correlations

Table 2 shows the correlation coefficients. When all groups were examined together, a statistically positive correlation was observed in levels of MPO, 8-OHdG in the gingival tissue, and H-score values of IL-6, TNF-α (p<0.001). Also, we found a positive correlation between gingival GSH levels and IL-10 H-score values (p=0.018). Furthermore, TNF-α and IL-10 H-score values were correlated with the alveolar bone level, alveolar bone area, and attachment level (p<0.001). In contrast, we found significant negative correlations between the TNF-α and IL-10 H-score values (p<0.001).

**Table 2:** The Spearman's rank correlation (r) among groups regarding MPO, 8-OHdG, GSH levels, H-Score values of TNF-α, IL-6, IL-10 and alveolar bone loss, alveolar bone area, and attachment level in all groups

		G_MPO	G_8OHdG	G_GSH	IL-10	IL-6	TNF-α	ABA	AL	ABL
G_MPO	r		.511**	-.155	-.285	.756**	.453**	-.269	.302	.265
	p		.001	.339	.075	.000	.003	.093	.058	.099
G_8OHdG	r	.511**		-.268	-.039	.411**	.283	-.051	-.048	-.032
	p	.001		.094	.810	.008	.077	.753	.768	.846
G_GSH	r	-.155	-.268		.372*	-.139	-.246	.137	-.227	-.213
	p	.339	.094		.018	.392	.125	.399	.160	.187
IL-10	r	-.285	-.039	.372*		-.270	-.649**	.784**	-.825**	-.765**
	p	.075	.810	.018		.093	.000	.000	.000	.000
IL-6	r	.756**	.411**	-.139	-.270		.514**	-.140	.292	.243
	p	.000	.008	.392	.093		.001	.389	.068	.131
TNF-α	r	.453**	.283	-.246	-.649**	.514**		-.605**	.601**	.549**
	p	.003	.077	.125	.000	.001		.000	.000	.000
ABA	r	-.269	-.051	.137	.784**	-.140	-.605**		-.693**	-.688**
	p	.093	.753	.399	.000	.389	.000		.000	.000
AL	r	.302	-.048	-.227	-.825**	.292	.601**	-.693**		.851**
	p	.058	.768	.160	.000	.068	.000	.000		.000
ABL	r	.265	-.032	-.213	-.765**	.243	.549**	-.688**	.851**	
	p	.099	.846	.187	.000	.131	.000	.000	.000	

** Correlation is significant at the 0.01 level.

*Correlation is significant at the 0.05 level.

G, gingival tissue; MPO, myeloperoxidase; 8-OHdG, 8-Hydroxydeoxyguanosine; IL, interleukin; TNF-α, Tumor necrosis factor-alpha; ABA, alveolar bone area; AL, attachment level; ABL, alveolar bone level.

## Discussion

Over the past decade, polyphenols have received increasing attention from researchers concerning human health, especially regarding dental caries and periodontal disease.^3^^,^^10^ The protective effects of polyphenols have been investigated in many animal studies^4,7–9^, however, few studies focusing on the therapeutic effects of polyphenols in periodontal lesions are available.^1^ In our study, we focused on the antioxidative and inflammatory effects of EA on periodontal repair related to experimentally induced periodontitis in rats and investigated their effects on the alveolar bone loss after EA administration. To the best knowledge, our study is the first to show that EA may provide curative effects on the periodontal tissues by reducing gingival oxidative stress and pro-inflammatory markers, and alveolar bone loss in the periodontal repair process after EP in rats.

In a previous study, daily oral administration of 7.5 mg/kg and 15 mg/kg EA effectively reversed abnormal changes in biochemical and hematological parameters in isoproterenol (ISO)-treated rats, returning them to near-normal levels.^26^ The study showed that the higher dose of EA (15 mg/kg) was more effective in restoring biochemical parameters to normal than the lower dose (7.5 mg/kg).^26^ In another study of Favarin, et al.^11^ (2013), they evaluated the anti-inflammatory effect of EA on acute lung injury and reported that 10mg\kg EA application had positive results on inflammatory cytokine levels. Since an effective dose has not yet been investigated in the treatment of periodontal diseases, in our study, we applied a 15 mg/kg dose of EA,^14^^,^^26^^,^^27^ reporting both its anti-inflammatory and anti-oxidant effects.

In our study, we induced experimental periodontitis for 14 days. Observational periods of 15 days or fewer are recommended in the EP model because rats have a large capacity to adapt to inflammatory stimulation.^2^^,^^21^ In our study, clinical symptoms such as edema, redness, bleeding, and mobility in the teeth were observed in the gingival tissues of the rats due to EP on the 14^th^ day following the ligature placement. Moreover, after the rats were euthanized on the 28^th^ day of study, the histomorphometric analysis showed that periodontitis was successfully induced in the experimental groups over the experimental 14-day period. These outcomes are in accordance with several previous studies.^2^^,^^21^ Herein, histologic assessments also showed greater degrees of alveolar bone resorption and periodontal tissue destruction in the rats of group 3 when compared with those of control groups. Nevertheless, our study demonstrated that rats treated with EA provided significant attachment and alveolar bone level gain. However, no previous studies have looked at the effects of EA on periodontal tissue destruction. The results of our study indicate that ellagic acid may reduce alveolar bone loss during periodontal repair after EP in the rat model.

Similar to previous studies,^28^^,^^29^ immunohistochemical results of our study showed that group 3 had the lowest IL-10 level and highest IL-6 and TNF-α levels in gingival tissues compared to the control groups. According to our findings, rats treated with EA (15 mg/kg) had significantly reduced IL-6 and TNF-α levels and increased IL-10 levels in gingival tissues. A previous review reported that ellagic acid reduced TNF-α and IL-6 levels *in vivo* and *in vitro*.^30^ A study has reported that TNF-α levels, which increase with the development of colonic inflammation, decreased after administration of EA.^31^ Another study observed that EA efficiently suppressed LPS-induced increased IL-6 levels depending on the dose.^6^ Moreover, Favarin, et al.^27^ (2013) stated that EA decreased IL-6 levels and elevated IL-10 levels in bronchoalveolar lavage fluid. Umesalma and Sudhandiran^32^ (2010) observed that EA regulates the inflammatory process by reducing the production of inflammatory cytokines through inhibition of NF-kB expression.^32^ Additionally, Usta, et al.^33^ (2013) suggested that the anti-inflammatory effect of EA may occur due to the suppression of cyclooxygenase (COX) protein activation. Granica, et al.^34^ (2016) stated that *Geum urbanum L.* roots, which is the rich source of EA derivatives, have a beneficial effect on gingival inflammation because it decreases TNF-α levels due to its effect on neutrophils and can be used in inflammation of mucositis, gingivitis, and periodontitis. Therefore, consistent with previous study findings,^6,11,30–32^ our results indicate that anti-inflammatory activity of EA in periodontium is evidenced by reducing proinflammatory cytokines and increasing anti-inflammatory cytokine activities in gingival tissue.

Several studies have proved that periodontal tissue loss is associated with increased reactive oxygen species and decreased antioxidant levels.^35–37^ Akman, et al.^38^ (2013) stated that MPO is the marker most commonly used for determining increased oxidative stress in periodontal diseases. Moreover, various studies found that rats with experimental periodontitis had high MPO activities in the gingival tissue compared to the control group.^28^^,^^29^^,^^38^^,^^39^ In accordance with these studies,^28^^,^^29^^,^^38^^,^^39^ our results showed that gingival tissue and serum MPO levels were higher in group 3 compared to the control groups. These results indicate that MPO activity measurement may be a useful marker for periodontitis. On the other hand, we determined that 8-OHdG levels, which are the markers of oxidative DNA damage, were higher in the gingival tissue and serum in group 3 compared to the control groups, although it was not statistically significant. Previous experimental studies have shown that 8-OHdG levels are positively correlated with periodontal tissue loss.^40^^,^^41^ The difference of our study may be related to the treatment with saline after ligature removal, improving the periodontal lesion and some reducing 8-OHdG levels. The findings of this study showed that, although no difference was observed in the control group treated with EA in the gingival tissue and serum MPO and 8-OHdG levels, these levels decreased in group 4 treated with EA when compared with the control groups. Moreover, analysis between experimental groups showed that EA significantly increased serum GSH levels. Many experimental studies have shown that EA significantly reduced the levels of MPO and increased the levels of GSH.^13^^,^^42^ Tomofuji, et al.^43^ (2009) stated that the production of ROS in the periodontal inflammation gradually reduced after ligature removal. Consistent with the study of Tomofuji, et al.^43^ (2009), our study indicated that EA administered systemically for 14 days had sufficient effect on the serum of experimental periodontitis rats but could not increase GSH levels in the gingival tissue adequately. Based on the results of this study, we deduce that EA may improve gingival oxidative stress and increase systemic antioxidant levels. However, there is no study in the literature to which we could compare our results.

However, our study has some limitations. The first limitation was the effect of EA on bone resorption based on the evaluation of biomarker levels and that the exact mechanism was not fully demonstrated. Second, ellagic acid levels were not evaluated in body fluids, so we could not determine how much systemic administered ellagic acid reached the gingival tissue. Third, our study is not a dose-dependent experimental study. Therefore, the effective and non-toxic dose of EA can be determined in periodontal tissues by experimental studies with different doses. Fourth, our study did not evaluate the effect of EA application on microbial dental biofilm in the periodontium. However, we reported that EA had anti-biofilm activity against bacterial pathogens.^7^ In addition to systemic administration, if EA was applied locally, EA’s anti-biofilm activity would be observed better in periodontal tissues. On the other hand, our study suggests that EA healed gingival oxidative stress and periodontal inflammation unconnected to the biofilm activity. Additionally, the experimental periodontitis model is not directly equivalent to chronic disease in humans. Future studies should further evaluate how EA administration affects gingival oxidative stress and periodontal inflammation in periodontitis patients.

## Conclusions

Our study showed that EA provides significant improvements on gingival oxidative stress and inflammatory markers and alveolar bone resorption in the periodontal repair process after experimental periodontitis within the limits of the study. These effects could be attributed to the antioxidative and anti-inflammatory nature of ellagic acid. Thus, EA may have therapeutic potential on periodontitis. However, studies should further explore and show the therapeutic mechanism of ellagic acid in periodontal inflammation and to examine its clinical effectiveness in human periodontal diseases, thus eliminating the limitations.
